# A Social Media Group Cognitive Behavioral Therapy Intervention to Prevent Depression in Perinatal Youth: Stakeholder Interviews and Intervention Design

**DOI:** 10.2196/26188

**Published:** 2021-09-15

**Authors:** Anupa Gewali, Alana Lopez, Kristin Dachelet, Elise Healy, Marimirca Jean-Baptiste, Holly Harridan, Yolanda Evans, Jennifer A Unger, Amritha Bhat, Darius Tandon, Keshet Ronen

**Affiliations:** 1 Department of Global Health University of Washington Seattle, WA United States; 2 Division of Adolescent Medicine Seattle Children's Hospital Seattle, WA United States; 3 Department of Obstetrics and Gynecology University of Washington Seattle, WA United States; 4 Department of Psychiatry and Behavioral Sciences University of Washington Seattle, WA United States; 5 Department of Medical Social Sciences Northwestern Feinberg School of Medicine Chicago, IL United States; 6 Center for Community Health Northwestern Feinberg School of Medicine Chicago, IL United States

**Keywords:** depression, mental health, perinatal, pregnancy, postpartum, adolescent, youth, social media, group, mobile phone

## Abstract

**Background:**

Adolescents and young adults aged <25 years (youth) are at a higher risk of perinatal depression than older adults, and they experience elevated barriers to in-person care. Digital platforms such as social media offer an accessible avenue to deliver group cognitive behavioral therapy (CBT) to perinatal youth.

**Objective:**

We aim to develop the Interactive Maternal Group for Information and Emotional Support (IMAGINE) intervention, a facilitated social media group CBT intervention to prevent perinatal depression in youth in the United States, by adapting the Mothers and Babies (MB) course, an evidence-based in-person group CBT intervention. In this study, we report perspectives of youth and health care providers on perinatal youths’ mental health needs and document how they informed IMAGINE design.

**Methods:**

We conducted 21 semistructured in-depth individual interviews with 10 pregnant or postpartum youths aged 14-24 years and 6 health care workers. All interviews were recorded, transcribed, and analyzed using deductive and inductive approaches to characterize perceptions of challenges and facilitators of youth perinatal mental health. Using a human-centered design approach, stakeholder perspectives were incorporated into the IMAGINE design. We classified MB adaptations to develop IMAGINE according to the Framework for Modification and Adaptation, reporting the nature, timing, reason, and goal of the adaptations.

**Results:**

Youth and health care workers described stigma associated with young pregnancy and parenting, social isolation, and lack of material resources as significant challenges to youth mental wellness. They identified nonjudgmental support, peer companionship, and access to step-by-step guidance as facilitators of youth mental wellness. They endorsed the use of a social media group to prevent perinatal depression and recommended that IMAGINE facilitate peer support, deliver content asynchronously to accommodate varied schedules, use a confidential platform, and facilitate the discussion of topics beyond the MB curriculum, such as navigating support resources or asking medical questions. IMAGINE was adapted from MB to accommodate stakeholder recommendations and facilitate the transition to web-based delivery. Content was tailored to be multimodal (text, images, and video), and the language was shortened and simplified. All content was designed for asynchronous engagement, and redundancy was added to accommodate intermittent access. The structure was loosened to allow the intervention facilitator to respond in real time to topics of interest for youth. A social media platform was selected that allows multiple conversation *channels* and conceals group member identity. All adaptations sought to preserve the fidelity of the MB core components.

**Conclusions:**

Our findings highlight the effect of stigmatization of young pregnancy and social determinants of health on youth perinatal mental health. Stakeholders supported the use of a social media group to create a supportive community and improve access to evidence-based depression prevention. This study demonstrates how a validated intervention can be tailored to this unique group.

## Introduction

### Background

Perinatal depression is defined as clinically significant depressive symptoms during pregnancy or up to 1 year postpartum. An estimated 13% of pregnant and postpartum women are affected by perinatal depression [1], with potentially significant effects on their psychological health, relationships with close family and friends, and the health of their children. Perinatal depression is associated with an elevated risk of preterm birth, insecure mother-infant attachment, and childhood emotional and behavioral problems [1-3]. Adolescents and young adults aged <25 years (youth) are at a higher risk of perinatal depression than older women (estimated prevalence 16-44% vs 10-20%) [4-7]. In 2017, an estimated 24.9% of births in the United States were to youth [8]. Although the frequency of adolescent pregnancy has declined over the last 25 years, marked racial and economic disparities persist, with significantly higher rates of pregnancy in youth of color than White youth [8]. These disparities are the result of intersecting inequities in access to financial resources, employment, education, medical care, and instrumental support, driven by structural racism and classism [9]. These forces mean that pregnant and postpartum youth experience multiple elevated stressors, compounded by their ongoing neurocognitive development.

Several interventions have been developed to prevent perinatal depression in adults. A recent US Preventive Services Task Force systematic review of intervention trials to prevent perinatal depression reported that counseling-based interventions were efficacious [10,11]. One of the counseling interventions identified by the report as supported by published studies is the Mothers and Babies (MB) course. Originally developed for low-income racial minority women in the United States, MB is based on cognitive behavioral therapy (CBT), a structured form of psychotherapy that focuses on identifying and modifying thought patterns and behaviors to interrupt their impact on feelings [12]. MB has been associated with a decline in depression incidence and depressive symptom severity in three randomized controlled trials and has been implemented at scale [13-17]. The course can be delivered in a group format, which cultivates mutual peer support, reduces stigma associated with mental health care, and is resource-efficient [13,18]. This feature also makes it ideally suited for use with youth, whose developmental stage is characterized by the growing influence of peer relationships and who may have especially limited social support [19-21]. Trials of MB have included youth participants, but no study has focused on evaluating its use exclusively in this age group.

Although in-person group counseling is a standard of care, it may be difficult for clients to access due to lack of transportation and child care, difficulty committing to a prespecified time to attend sessions, and experienced or internalized stigma of seeking mental health care [21,22]. Clinic-based care is often designed for the needs and expectations of privileged adult populations, so these barriers may be most acute among youth, whose neurocognitive development and societal position limit their engagement in clinic-based care [23]. In addition, the COVID-19 pandemic and consequent physical distancing protocols have created enormous disruptions in in-person mental health care, while disproportionately disrupting the economic stability, social connection, and mental health of women, children, and adolescents, particularly in low-income communities and communities of color [24].

Technological applications such as social media (interactive forms of web-based content and communication) provide an opportunity to overcome access barriers to in-person services [25,26]. Several internet-based CBT interventions have been developed and applied to perinatal depression, including electronic adaptation of the MB course, but most have not leveraged the benefits of group delivery, and none have targeted youth [26-34]. Social media offers an underexplored and accessible avenue through which to deliver group CBT to perinatal youth; as of 2018, 94% of those aged 13-17 years in the United States used social media and 95% had a smartphone [35].

We adapted the evidence-based MB course from a 6-session in-person group intervention to a 12-week social media group intervention tailored for youth aged <25 years. The resulting intervention, named the Interactive Maternal Group for Information and Emotional Support (IMAGINE), is being evaluated in an ongoing pilot study. Our adaptation process used a human-centered design approach [36], focusing on modifications to MB that were needed to (1) meet the unique mental health needs of perinatal youth and (2) deliver content through a digital delivery modality. The adaptation of evidence-based interventions, defined as thoughtful and deliberate alteration of their design or delivery to improve fit or effectiveness [37], is an important process that frequently accompanies *real-world* implementations. Some adaptations may enhance intervention effectiveness, whereas others may undermine it. Detailed documentation of adaptations is therefore crucial for interpreting data on intervention efficacy and effectiveness and understanding the core functions of evidence-based interventions. The Framework for Modification and Adaptation (FRAME) was developed as a structure for systematic reporting of modifications in terms of what was modified (context, content, or training), the nature of the modification, who made the modification, when in the process of implementation was the modification done, the reason and goal of the modification, and whether the modification was fidelity-consistent [37,38].

### Objectives

In this paper, we aim to present findings from formative stakeholder interviews regarding perinatal youths’ mental health challenges, facilitators, and design recommendations for a social media intervention to prevent perinatal depression. On the basis of these formative findings, we aim to describe the adaptations made to MB to develop IMAGINE, systematically reporting our modifications according to the FRAME.

## Methods

### Study Design

This paper documents the development of the IMAGINE intervention, a social media group CBT intervention tailored for perinatal youth. Two sources of formative data were analyzed: (1) in-depth interviews (IDIs) with perinatal youth and health care providers exploring youths’ perinatal mental health experiences and their design recommendations for a social media intervention and (2) study memos documenting adaptations and refinements made to the MB course to target youth and be delivered by social media. Study memos were reflexively composed by the study team after both rounds of IDIs and intervention development was completed to document the team’s own decisions.

### Study Population and Participant Recruitment

Two rounds of IDIs were conducted between November 2019 and July 2020. The first included six IDIs with perinatal youth and six IDIs with health care providers who care for perinatal youth. The second round included IDIs with nine perinatal youths, five of whom were also participants in the first round. Youth participants were purposively recruited through in-person outreach at health care facilities and community events and through targeted advertisements on Instagram and Facebook in Washington State. In round 2, Instagram and Facebook advertisements were expanded throughout the United States. Health care providers throughout the United States were purposively recruited via email and phone through the professional networks of the study team.

Eligible youth were pregnant or ≤2 years postpartum, aged 14-24 years during pregnancy, had access to a smartphone, and spoke English. These criteria were selected to reflect the target population for the IMAGINE pilot study. Although perinatal depression is defined as occurring up to 1 year postpartum, youth who were up to 2 years postpartum were included to allow participants to reflect on the entire period of risk for perinatal depression. Eligible providers were aged ≥18 years and worked as nurses, physicians, social workers, or mental health care workers (HCWs) with perinatal youth.

### Data Collection

The first round of IDIs was conducted either in-person in Seattle, Washington, or via phone, per participant preference. The second round occurred after the COVID-19 restrictions were instated, and all interviews were conducted by phone or videoconference. IDIs were conducted in English and included a demographic questionnaire, administered electronically using REDCap (Research Electronic Data Capture; Vanderbilt University), followed by a semistructured discussion guide exploring literature-based determinants of perinatal depression and elements of the MB intervention. All IDIs were audio recorded and transcribed.

Round 1 IDIs discussed expectations, challenges, and support during and after pregnancy, as well as the need for additional support. After outlining a hypothetical virtual group intervention, interviews explored different content areas of interest and social media platform functionality, content delivery, and considerations about coparticipants and facilitators. A rapid content analysis was performed to identify key themes after the first round of IDIs, and the results were used to inform design and social media platform selection for an IMAGINE prototype. The second round of IDIs explored the impact of COVID-19 on mental well-being and included a live, interactive demonstration of the IMAGINE prototype presented by screensharing over videoconference to elicit detailed participant feedback. Rapid analysis of round 2 IDIs was used to refine the IMAGINE content.

The study team prepared memos documenting the team’s design discussions and decisions during the adaptation of the MB course to develop IMAGINE. Publicly available MB materials were used as the starting point, with some material drawn from an online version, e-MB [29,39]. Adaptation occurred through a series of collaborative meetings, including the study principal investigator (KR), research assistant (AG), intervention facilitator (KD), and community member advisor (MJB). Additional input was sought from other coauthors, including a researcher who led previous MB trials (DT).

### Data Analysis

We conducted a descriptive content analysis of all 21 IDIs, focusing on characterizing perinatal youth’s mental health challenges, facilitators, and the potential role of a social media intervention. Authors AL, AG, and KR generated an initial codebook for IDIs that included deductive codes based on the literature and inductive codes based on a review of a subset of transcripts. Study team memos were also coded using a second codebook based on the domains of the FRAME to categorize the decisions made and reasoning given by the research team [37]. Following the initial development of both codebooks, a consensus coding process was carried out by AL, AG, and KR and modifications were made to each codebook and code definition until all parties agreed. Following consensus coding, all transcripts and study team memos were coded independently by AL or AG and reviewed by another team member. Disagreements were resolved through group discussions with KR. All coding was conducted using Dedoose (version 7.0.23, SocioCultural Research Consultants, LLC). Coded excerpts were organized and synthesized into thematic memos and discussed by AG, AL, and KR.

### Ethical Considerations

The study was reviewed and approved by the institutional review board of the University of Washington. All participants provided informed consent. Waivers were obtained for written documentation of informed consent and parental consent for adolescents aged <18 years.

## Results

### Characteristics of Study Participants

The sociodemographic characteristics of the youth and HCW participants in the formative interviews are summarized in Table 1. A total of 15 interviews were conducted with 10 youths and 6 interviews were conducted with HCWs. Youths interviewed in round 1 resided in Washington State and had a median age of 22 years (IQR 21-23). All round 1 participants identified as people of color. In total, of the 6 participants, 2 (33%) were currently pregnant, and the rest had children of a median age of 5 months. Most (5/6, 83%) had some experience with mental health counseling. Youths interviewed in round 2 resided in Washington, Michigan, and Texas and had a median age of 21 years (IQR 20-23). All round 2 participants identified as people of color. In total, of the 9 participants, 2 (22%) were currently pregnant, and the rest had children of a median age of 7 months. Most (7/9, 78%) had some experience with mental health counseling. HCWs were mostly (4/6, 66%) physicians, 1 nurse, and 1 physician’s assistant, working in Washington, Colorado, and Rhode Island. They had been working for a median of 6 years in their profession (IQR 2-27) and saw a range of <10 to >100 perinatal youth participants annually.

**Table 1:** Participant characteristics.

Characteristic			Values
**Youth round 1**			
	Age (years; n=6), median (IQR)		22 (21-23)
	**Race^a^** **(n=6), n (%)**		
		African American	2 (33.3)
		American Indian or Alaska Native	1 (16.7)
		Asian	1 (16.7)
		Latinx	3 (50)
		Native Hawaiian or Pacific Islander	0 (0)
		White	1 (16.7)
		Other	2 (33.3)
	Primiparous (n=6), n (%)		1 (16.7)
	Currently pregnant (n=6), n (%)		2 (33.3)
	Age of youngest child (months; n=5), median (IQR)		5 (0-5)
	History of depression diagnosis (n=5), n (%)		1 (20)
	History of mental health counseling (n=6), n (%)		5 (83.3)
	**State** **(n=6), n (%)**		
		Washington	6 (100)
**Youth round 2^b^**			
	Age (years; n=9), median (IQR)		21 (20-23)
	**Race^a^** **(n=9), n (%)**		
		African American	2 (22.2)
		American Indian or Alaska Native	0 (0)
		Asian	2 (22.2)
		Latinx	5 (55.6)
		Native Hawaiian or Pacific Islander	0 (0)
		White	1 (11.1)
		Other	1 (11.1)
	Primiparous (n=9), n (%)		1 (11.1)
	Currently pregnant (n=9), n (%)		2 (22.2)
	Age of youngest child (months; n=8), median (IQR)		7 (3-17)
	History of depression diagnosis (n=8), n (%)		6 (75)
	History of mental health counseling (n=9), n (%)		7 (77.8)
	**State** **(n=9), n (%)**		
		Washington	7 (77.8)
		Michigan	1 (11.1)
		Texas	1 (11.1)
**Health care worker**			
	Age (years; n=6), median (IQR)		42 (37-58)
	**Profession** **(n=6), n (%)**		
		Nurse	1 (16.7)
		Physician	4 (66.7)
		Physician’s assistant	1 (16.7)
	Years in profession (n=6), median (IQR)		6 (2-27)
	**Pregnant patients aged <25 years seen in last year** **(n=6), n (%)**		
		<10	1 (11.1)
		10-50	2 (33.3)
		51-100	0 (0)
		>100	3 (50)
	**State** **(n=6), n (%)**		
		Washington	2 (33.3)
		Colorado	3 (50)
		Rhode Island	1 (11.1)

^a^Race categories are not mutually exclusive.

^b^Five round 1 participants were also interviewed in round 2.

### Challenges Contributing to Youth Perinatal Depression: Stigma, Isolation, and Lack of Material Resources

**Figure 1:**
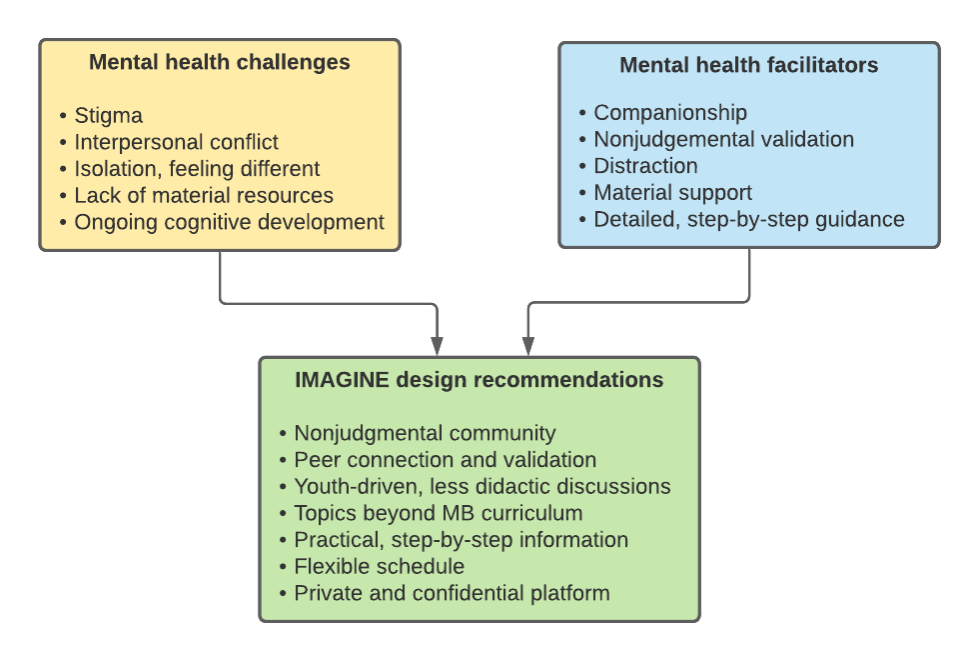
Summary of youth and health care worker interview themes regarding challenges to and facilitators of youth perinatal mental health and Interactive Maternal Group for Information and Emotional Support design recommendations. MB: Mothers and Babies, IMAGINE: Interactive Maternal Group for Information and Emotional Support.

Figure 1 summarizes the factors identified by the youth and HCWs as challenges to youths’ mental wellness, as well as the factors participants described as enabling youth mental wellness and the resulting IMAGINE design suggestions they offered (described in detail in the sections below on facilitators and intervention adaptation). Overall, the youth and HCWs highlighted similar themes.

Participants frequently identified stigma associated with young pregnancy and parenthood as a significant challenge to their mental health. They described family, friends, and religious communities as sources of judgment and harsh criticism in pregnancy and the postpartum period. The youth described losing material and emotional support and, in some cases, losing relationships entirely as a result of their pregnancy, leading to feelings of loneliness, humiliation, and self-doubt:

I had to actually change schools because when people started finding out about my pregnancy, they began to say some, let’s just say, very disparaging things. Like “you shouldn’t have kids. You’ll be a terrible mother. I feel bad for that baby. She deserves better.” And just a whole bunch of very mean and negative things that made me very depressed...Listening to one of your closest friends say that you shouldn’t have kids or that you’re gonna make a terrible mother is a very hard thing to hear because you need support at that time.

Youth participant 1008

In relationships that continued through pregnancy and childbirth, the youth and HCWs highlighted considerable stress associated with negotiating new relationship roles and dynamics. Interpersonal conflicts were frequently mentioned, with the youth’s family of origin, partners, coparents, friends at school, and friends on social media. In particular, participants reported their parenting decisions being judged and questioned, which led to feelings of guilt and internal conflict. For example, one participant felt immense relief after finally securing a job and starting to pay bills after her delivery but struggled to balance this with the cultural stigma she felt from her family, who believed she should be caring for her baby rather than working:

My parents are from Mexico and there’s a lot of...stigma, like, being there with your...child the whole time and not taking them in daycare. [My family said that] I started work too soon...I’m not taking care of her.

Youth participant 1004

Similarly, negative interactions with HCWs in which the youth felt disregarded were reported to contribute to feelings of shame and postpartum depression. One participant described a traumatic interaction with a physician during delivery, impacting her mental health for months afterward:

I was 19? 20? when I gave birth and so I wasn’t really sure how to advocate for myself and what I wanted from people, especially in the delivery room. I had a pretty uncomfortable experience with the male provider in charge of my labor...and then I ended up having an emergency C-section. And then...the first four months I was really unable to connect with my kiddo just because of how traumatic the birth was, and getting over C-section, and being young and confused...I felt something was wrong with me because I couldn’t connect with my child and feel that automatic love and connection.

Youth participant 1006

Many participants felt that they would have been more respected if they were older and commented on intersectionality with their race and ethnicity. One participant reported that her assessment of her due date and last menstrual period was ignored:

I don’t know why, maybe [because of] my ethnicity. Maybe, I don’t know, my age.

Youth participant 1004

HCW participants themselves also identified providers as sources of stigma and emotional distress:

There is a lot of stigma associated with being a pregnant youth or young parent. Stigma from peers, stigma from family, stigma from community, and stigma from health care providers. This has major implication for mental health.

HCW participant 4001

Youth participants commonly reported feeling *different*, both from age-mates, who typically did not have children, and from older mothers, who did not experience the same level of stigma and appeared to have more resources. This difficulty in connecting with others contributed to the isolation and lack of emotional support:

[B]eing a young mom there is a lot of, not necessarily shame, but a little bit of stigma and difficulty in my ability to interact with people my age because...at 19, people aren’t usually pregnant or raising children or worried about bills and household and all. But for me...I always felt like, OK, I spend this much time out of the house and away from my kid, and then I have to work on top of that. And then I go home and I do homework, so [my daughter] is not really getting the attention that she needs from me, so I can’t justify going out with friends. So I haven’t done that in years.

Youth participant 1006

Participants argued that these relational and emotional challenges were heightened by the youths’ lack of material resources. Several youths reported that the need to earn money and lack of childcare support meant they had very little time to attend to their emotional or physical needs, such as eating or sleeping, and that these needs were secondary to caring for their child. HCWs and youth also highlighted that these challenges were exacerbated by their *growing up early* and navigating a transition to parenthood while still developing their own cognition and sense of self:

I was so young when I had my first daughter, I was 14...I’m just now starting to talk like I’m a teen again...just now starting to figure out what I want...how I actually want to dress...instead of just doing what I can because I have to focus on my kids first.

Youth participant 1005

Being responsible for another human being on an ongoing basis all the time: that’s a big change. Negotiating the place in their family...whereas before they’re a teenager in the family and now suddenly they’re the mother of a child and teenager in the family, that’s a big problem. And then there’s financial issues. Actually taking care of the child and getting finances together. And then there’s social challenges, learning how to interact with their peers and find their own place just as an individual.

HCW participant 4002

In the youth IDIs conducted during the COVID-19 pandemic, participants discussed COVID-19 and related restrictions as additional challenges, for example, by hampering previous methods of stress relief, undermining therapeutic relationships with HCWs, and increasing anxiety.

### Facilitators of Perinatal Mental Wellness in Youth: Nonjudgement, Material Support, and Distraction

Figure 1 summarizes the themes that the youth and HCWs identified as supportive of youth perinatal mental wellness. The youth consistently spoke of nonjudgmental support and companionship from others with shared experience as a crucial facilitator of their mental wellness. Three youth respondents had attended in-person support groups for young mothers and highlighted the friendships formed through these groups as especially important. In these interactions, they felt affirmed and could bond over shared experiences with other group members, free of stigma:

I would say [the only non-judgmental adults] that are in my life [are] my mommy groups, just because, you know, they’re going through what I’m going through, they went through something worse of what I went through.

Youth participant 1002

Furthermore, the youth identified that their peers’ shared experiences rendered their advice more credible and relevant than advice from health care providers:

Some doctors, they don’t go through...the things they see people go through...Doctors could be like 30 and have a kid, and [the people in my support group] were like 19 or 20 that had to deal with negativity...[They] would just basically tell me how they dealt with depression and how they dealt with insecurity and stuff like that. So I just went off of what they did.

Youth participant 1010

Similarly, some HCWs commented that although many in-person groups centered around perinatal care, peer-to-peer connections were more difficult to find and a crucial source of positive reinforcement that mothers could call upon throughout pregnancy and afterward. When prompted, many youths shared that they found supportive *online friends* through informal social media groups or perinatal support mobile apps, who provided honest advice, validation, and companionship. One postpartum participant reported that the act of helping others by posting in an online group lifted her spirits and supported her own mental wellness.

Youth participants argued that access to material support and childcare were important facilitators of mental wellness. Several reported that they accessed assistance programs for new parents, and some had a health care provider with whom they felt very close and who provided support and information. However, some participants reported that although they knew that support was available, they did not always feel confident in navigating the details. This highlighted detailed, concrete guidance as an often-unmet need:

[I wish] someone would inform me about events, activities...WIC tells me about birthing classes, like “you should look into that”. But I’m like, “Okay, where do I start? Who do I ask?” That information is given, but not someone that can help you with specifics.

Youth participant 1003

Several youths, especially those who were pregnant at the interview, named a variety of distractions and stress reduction methods they used to support their mental wellness, including listening to music, exercise, digital entertainment, or going to a salon. Several postpartum participants identified their children as a source of motivation and support: one participant stated that caring for her baby kept her present and focused and another highlighted that her children gave her a renewed sense of purpose and drive to accomplish goals.

### IMAGINE Design Recommendations to Support Youth Mental Wellness

To emulate the facilitators identified earlier, the youth recommended that a virtual program such as IMAGINE provides a space to connect with peers and discuss members’ specific concerns and circumstances. They suggested that IMAGINE addresses youth-driven topics beyond the MB depression prevention curriculum:

The one thing that most pregnant people who are by themselves need is just somebody to listen to them. Because it’s very easy to feel overwhelmed and unheard of when people around you are speaking such negative comments. So if [I] can just...have this group that I go to and I can talk about what I feel. I can ask as many questions as I want, and they will give me positive feedback and they will let me know whatever information I need so I can make myself a better person. That would have been very helpful.

Youth participant 1005

Some youths described positive experiences with online forums where multiple conversations about different topics could take place in parallel *channels*:

Let’s say that I had a bad day, for instance. And I just kinda wanted to get some positive vibes from people who I was engaging with over the last few weeks...It would be really cool to have, like, a vent/get positive signs type chat room.

Youth participant 1012

HCWs similarly suggested that the amount of structured content could be reduced to create more flexibility and space for youth-driven discussions:

What resonates with [youth] are human interactions where they can tell their story, where they feel like other people care for them and where they learn in a more subtle fashion than somebody sort of didactically telling them what to do.

HCW participant 4002

Consistent with the material challenges listed earlier, HCWs and youths described difficulties in attending appointments at fixed times due to busy and unpredictable schedules. Participants were therefore enthusiastic about asynchronous content or discussions that could be accessed and caught up on whenever they had a break, such as when their child was asleep.

The privacy of a social media group was discussed by many participants. HCWs reported more concern than youth participants and suggested that clear norms regarding confidentiality be established and continually reinforced by the group facilitator. When prompted, youth participants agreed that social media groups could breed hostility and confidentiality breaches but said they would feel comfortable engaging if options were provided to conceal one’s identity, for example, making photos or video-based contributions optional:

Confidentiality would depend on the rules of the game and everyone participating, understanding those rules and standing by them...Confidentiality issues are a little bit trickier on the online platform and what your securities are.

HCW participant 4005

You know how on iPhones you can, like, screen record? Not saying that this is going to happen at all. But someone who is a part of [the group] can screen record and put someone’s personal business out.

Youth participant 1001

### Adaptation of the MB Course to Create the IMAGINE Intervention

The abovementioned stakeholder input was used to inform the adaptation of MB course materials by the study team and documented in the study team memos. In the following section, we describe the final IMAGINE intervention and outline the adaptations made in developing it.

#### Overview of the IMAGINE Intervention

**Figure 2:**
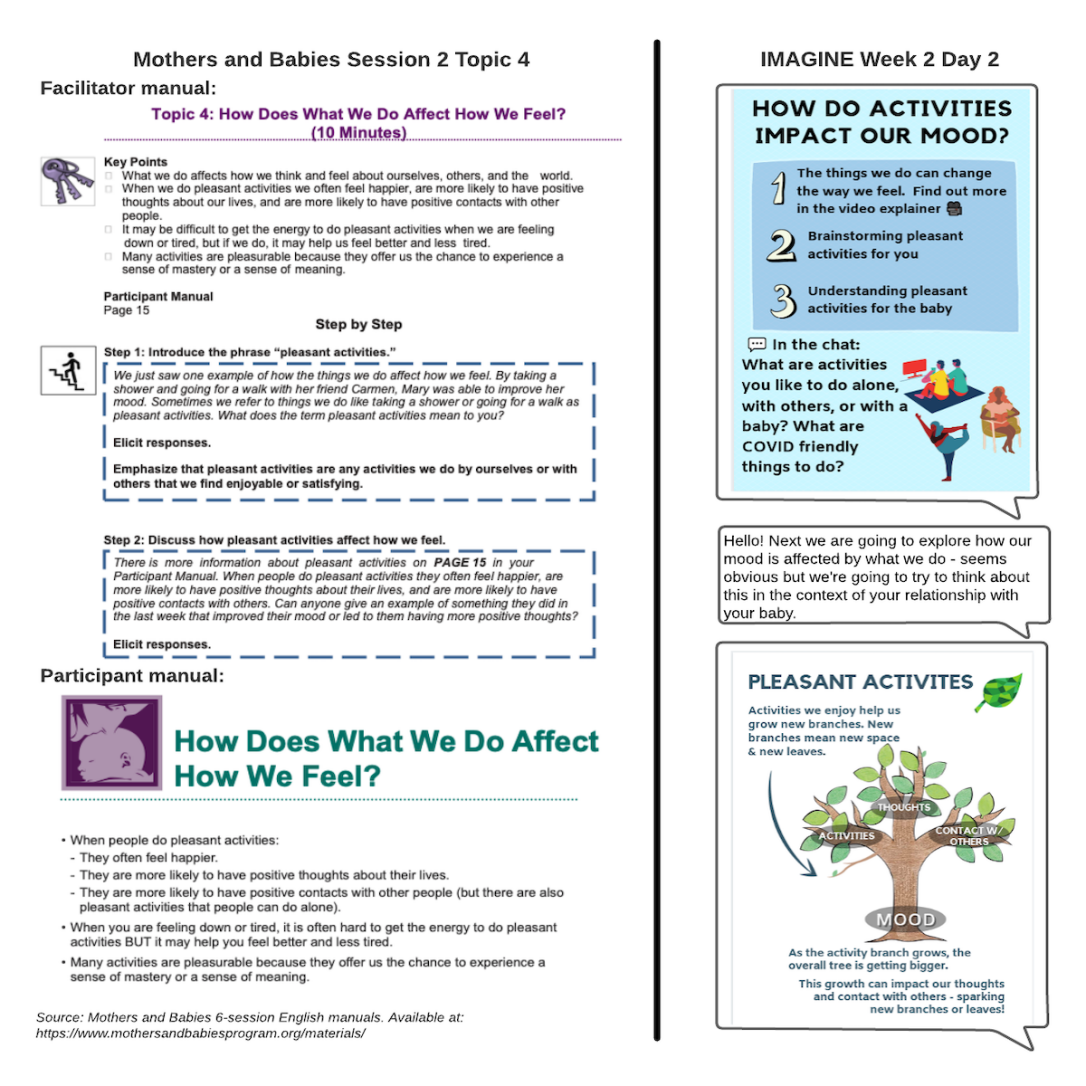
An example of Mothers and Babies and adapted Interactive Maternal Group for Information and Emotional Support content.

Figure 2 illustrates the example content in the MB manual compared with the corresponding messages in IMAGINE. The IMAGINE intervention consists of groups of 6-10 perinatal youths on the messaging platform, Slack, facilitated by a study social worker. The group facilitator delivers adapted MB course content through daily communication, including text messages, informational graphics, and short prerecorded *selfie* videos of the facilitator. Facilitator communication is intended to promote group discussions and personal reflections. Once per week, the facilitator holds a group video call, in which participants are encouraged but not required to attend. All content can be engaged asynchronously, and the material discussed in the video call is also summarized in a message. The Slack group contains five *channels* for different communications. The *#daily-content* channel contains the facilitator’s daily MB content and resulting participant discussions, as well as summaries of weekly video calls. The *#reference-materials* channel contains visual summaries shared in the *#daily-content* channel as the intervention proceeds, to act as reference materials for future review. The *#community-conversations* channel allows participants to discuss topics of their choosing that are unrelated to MB content. The *#ask-an-expert* channel is intended for participants to submit questions when they want detailed information or guidance from someone with specialized training, such a midwife, therapist, or social worker. The facilitator is responsible for working with a multidisciplinary team to address these questions. Finally, the *#personal-project* channel is a private channel to which only individual participants and the facilitator have access, in which the participant answers reflection questions three times per week regarding their mood and CBT activities. In addition, participants are able to send a private direct message to other participants and to the facilitator. Participants can also choose how they are identified in the group: Slack accounts are identified by email address, and there is no requirement to create a username that reflects the participant’s name.

#### Summary of Adaptations

Table 2 summarizes the major adaptations made to MB in producing IMAGINE, categorized according to the elements outlined in the FRAME. The goal of all adaptations was not to revise core intervention domains hypothesized to lead to mental health outcomes but rather to modify components of the intervention’s *adaptable periphery* [40]. All adaptations were therefore intended to be fidelity-consistent.

**Table 2:** Summary of adaptations made to the Mothers and Babies course in developing Interactive Maternal Group for Information and Emotional Support, classified according to the Framework for Modification and Adaptation.

Adaptation	Classification	Reasons	Goal	Timing	Participant recommendation addressed
Asynchronous, virtual delivery through the Slack messaging platform	*Context*^a^: format	*Recipient*: increased barriers to in-person and synchronous care among young people *Recipient*: limited access to resources necessitates free, phone-compatible platform *Recipient*: Slack compatible with functionality preferences identified in formative interviews	Improve fit with recipients Increase reach	A priori conceptualization After formative interviews	Flexible schedule Confidential platform Topics beyond MB^b^ curriculum
Multimodal communication (graphics, text messages, recap videos, and optional video calls); redundant content	*Context*: format *Content*: tailoring *Content*: repeating	*Recipient*: multimedia content appeals to youth *Recipient*: limited time and access to technology mean intermittent engagement and necessitate redundancy *Setting*: virtual platform necessitates varied content to avoid participant overwhelm *Provider*: metaphors and exercises used in facilitator’s prior work with youth provided alternative materials	Improve fit with recipients Improve effectiveness	After formative interviews	Flexible schedule Youth-driven, less didactic discussions
Shorter, simplified language	*Content*: shortening	*Recipient*: short, simple text better understood by youth *Setting*: visual delivery necessitates many short messages rather than fewer longer messages	Improve effectiveness	A priori conceptualization	—^c^
Conversion of six 2-hour sessions to 10-12 weeks	*Content*: lengthening, spreading	*Setting*: visual delivery necessitates many short messages rather than fewer longer messages	Improve fit with recipients Improve feasibility	A priori conceptualization	—
Increased emphasis on encouraging peer participation; addition of *channel* for unstructured peer discussions and practical advice	*Content*: tailoring *Content*: adding	*Setting*: virtual platform increases challenge of creating connection *Recipient*: developmental stage and stigma make peer support especially powerful and didactic *school-like* content especially unappealing	Improve effectiveness	After formative interviews	Peer connection and validation Topics beyond MB curriculum Practical, step-by-step information
Breaks and pauses in content for discussion; adaptable timing based on participant feedback	*Context*: format *Content*: loosening structure	*Setting*: asynchronous platform increases risk of losing participant engagement and limits enforcement of regular participation *Recipient*: youths’ experiences of dismissal and stigma drive desire for youth-driven content	Improve fit with recipients Improve effectiveness	After formative interviews	Flexible schedule Youth-driven, less didactic discussions
Emphasis on normalizing challenges; ensuring exercises not viewed as judgmental	*Content*: tailoring	*Recipient*: youths’ experiences of dismissal and stigma drive need for validation and normalization	Improve fit with recipients Improve effectiveness	After formative interviews	Peer connection and validation
Incorporation of COVID-19 as a stressor and modifier of activities	*Content*: adding	*Sociopolitical*: COVID-19 is a new stressor and modifier of resilience strategies	Improve fit with recipients	After formative interviews	Practical, step-by-step information
Removing assigned educational video and role-playing	*Content*: skipping	*Setting*: asynchronous platform made role-playing impractical *Recipient*: 15-minute educational video viewed as unappealing to youth	Improve fit with recipients Improve feasibility	After formative interviews	Youth-driven, less didactic discussions
Mood assessment three times per week rather than daily	*Content*: skipping	*Setting*: need for reduced message volume on virtual platform	Improve fit with recipients Improve feasibility	After formative interviews	Flexible schedule
Participants are located all over the United States	*Context*: setting	*Setting*: virtual delivery facilitates remote recruitment *Setting*: study timeline requires parallel recruitment in multiple sites	Improve feasibility	After formative interviews	—

^a^Italics indicate Framework for Modification and Adaptation elements.

^b^MB: Mothers and Babies.

^c^The adaptation did not address a participant recommendation.

The most significant adaptation, which motivated many other adaptations, was modifying the context from in-person to virtual delivery. This change was a central premise of the IMAGINE study, driven by the goal of increasing reach and improving fit with youth recipients who are not well served by in-person or synchronous care. Formative interviews confirmed that attending meetings at fixed times was challenging for many youths, so all content was designed to be accessed asynchronously. The Slack platform was selected for intervention delivery based on its ability to fulfill desired functionalities discussed in formative interviews, specifically having multiple parallel conversation *channels*, individual messaging with the facilitator, and nonidentifiability. In the second round of formative interviews, youth participants universally reported that the platform was acceptable.

The use of virtual, asynchronous delivery necessitated additional adaptations to the context and content of the intervention. On the basis of youth and HCW recommendations that the intervention be youth-driven and less didactic, the study team determined that delivering all MB content through text messages would create a large message volume that would quickly lead to recipient disengagement. In order to improve fit with recipients and therefore improve effectiveness, MB content was summarized into short text messages using simplified language and spread from six 2-hour sessions into multiple daily messages over 10-12 weeks. To accommodate different engagement styles, multimodal visual content was developed, including graphical summaries of complex concepts, 1- to 3-minute informal videos of the facilitator summarizing materials, and optional weekly synchronous video calls. Materials deliberately included some redundancy so that recipients who did not access the intervention daily or were unable to attend synchronous video calls could still remain up to date. An additional reason for developing visual and simplified content was that youth recipients’ neurocognitive and educational stages made short multimedia content especially appropriate.

In response to recommendations for a flexible intervention and youth-driven discussion (Figure 1), a looser, more adaptable structure was used, with intentional breaks in content for discussion, as well as active solicitation of participant feedback during the intervention. In view of youths’ experiences of stigma and requests for nonjudgmental and affirming support, examples were examined carefully and tailored to normalize mental health challenges and avoid content that could be perceived as judgmental. Several additions were made to content to improve fit with the youth’s stated preferences, including having multiple parallel *channels* where participants could have an unstructured conversation about topics not included in the MB curriculum and receive practical step-by-step guidance on their own questions. Structured, planned facilitator content was minimal in these channels; the goal was to provide a forum in which participants could ask questions of each other or the facilitator and share advice with each other. COVID-19 was additionally incorporated throughout the content to acknowledge its impact as a stressor and barrier to certain pleasant activities or contact with others. To improve feasibility online and reduce didactic content, some activities, including an educational video and role-plays, were removed, and daily mood checks were reduced in frequency to three times per week. Finally, for reasons of feasibility, the pilot study recruits participants throughout the United States, and youth from multiple locations will be in the same virtual group.

## Discussion

### Principal Findings

In this manuscript, we presented the perspectives of youths and health care providers in the United States on perinatal youths’ mental health challenges, resources, and design recommendations for a social media intervention. We also reported how we incorporated these perspectives and recommendations into the development of IMAGINE by adapting the MB course for virtual delivery, tailored for youth aged <25 years.

We found that the youth and HCWs identified stigma, isolation, and lack of material resources as significant challenges to their mental wellness. Stigma associated with young pregnancy and parenting was described as leading to interpersonal conflict and a lack of community support, fueling feelings of guilt and shame. Poverty and lack of time for self-care compounded these challenges, and COVID-19 presented a new stressor and barrier to resilience activities.

The youth and HCWs also identified a number of facilitators of perinatal mental wellness and highlighted ways to augment these through IMAGINE. Relational factors were identified as supportive of perinatal mental wellness, with validation, companionship, and peer support highlighted as critical means by which a virtual group could counteract the stigmatization and isolation many reported as challenges. Practical support, such as access to information and step-by-step guidance, was also identified as a desired function that could be delivered through a virtual communication platform.

The recommendations made in our formative interviews led to several adaptations to the content and context of MB to develop IMAGINE. The goals of the adaptations were to improve fit with recipients, improve effectiveness, increase reach, and improve feasibility. All adaptations were fidelity-consistent, based on consultation with a developer of the MB course. Content adaptations were mostly tailoring content and loosening structure, with a few additions and reductions in frequency. The reasons for the adaptations were motivated mostly by recipient characteristics and recommendations shared in formative work, such as elevated barriers to synchronous care, limited access to resources, stigmatization, and developmental appropriateness of written versus visual material. Many adaptations have been made to accommodate virtual delivery.

### Comparison With Prior Work

Qualitative accounts of perinatal youths’ own perspectives on contributors to their mental wellness are critical to inform responsive and effective care. Findings from our formative interviews add to the literature describing perinatal youth experiences [41,42]. Consistent with our findings of challenges and facilitators of perinatal mental wellness and the interplay between them, a recent systematic review and meta-ethnography reported themes at the individual, relational, societal, and economic levels shaped youths’ mental well-being [41]. Collectively, these findings highlight the impact of the stigmatization of young pregnancy and the importance of addressing interpersonal relationships and social determinants of health to improve youth perinatal mental health [43,44].

In addition to providing insights into perinatal youths’ experiences, our study reports recommendations to inform the development of a social media CBT intervention. Several studies have tested internet or mobile app delivery of individual-level CBT for perinatal depression and found promising results [26,27,29-34]. However, only one virtual intervention was designed for delivery in a group format [30,31], and to our knowledge, none have focused specifically on perinatal youth. Our study therefore provides novel data to inform the design of social media interventions for perinatal youth mental health. Two previous interventions have adapted the MB course for virtual delivery. Barrera et al [29] adapted the program as a self-guided individual internet program that included videos, written materials, and visual summaries. Consistent with our findings, the intervention of Barrera et al [45] recommended simplification of content, detailed instructions, and graphical representations. Sawyer et al [30,31] also used the MB content as part of a mobile app. However, the approach used to adapt the MB curriculum for virtual delivery in this intervention has not been reported. Our use of formative interviews to drive a human-centered design process provided unexpected design recommendations, such as the use of a platform that allowed multiple parallel conversations, flexibility in timing and medium of communication, and provision of practical step-by-step information.

The process of adaptation is increasingly recognized as an important determinant of the effectiveness of evidence-based interventions, with implementers often weighing the importance of maintaining intervention fidelity against the need to adapt delivery for a specific context. Our structured reporting of adaptations according to the FRAME contributes to a growing body of literature that systematically reports adaptation processes [46,47]. To our knowledge, this study is the first to apply this approach to the virtual adaptation of an in-person intervention and to the adaptation of an intervention by a research team *before* its implementation. This approach can be used by others planning the adaptation of an evidence-based intervention to ensure that modifications are well-justified and provide documentation to inform the interpretation of effectiveness data.

### Limitations

Our findings should be interpreted in light of the limitations of the study. First, our study population was recruited through convenience sampling, and although participants from across the United States were included, our sample size was small and Washington State was overrepresented. In addition, we recruited several youth participants through health care facilities and community programs, which may have preferentially captured the perspectives of youth who had relatively high engagement in existing services, which may explain the high proportion (approximately 80%) of youth participants who had experience with mental health counseling. Our evaluation of the intervention adaptation process was reflexive. We assessed our own changes, so it is possible that we omitted some changes that would have been noted by an external reviewer or misclassified our motivation for making them. Finally, when making adaptations, we sought guidance from one of the developers of the MB course in an effort to preserve fidelity. However, data on which elements of the course are core for efficacy are limited, and we have not conducted a formal assessment of fidelity [48]. The impact of our adaptations on intervention efficacy will need to be empirically tested as part of IMAGINE’s evaluation.

### Conclusions

In conclusion, our study provides an in-depth assessment of the mental health needs and assets of perinatal youth and systematic documentation of adaptation of the MB course for digital delivery in response to youth’s context and recommendations. Our study highlights the potential of social media group interventions to support perinatal youth mental wellness and provides an example of how existing intervention materials can be tailored to this unique group.

## Abbreviations

CBT: cognitive behavioral therapy
FRAME: Framework for Modification and Adaptation
HCW: health care worker
IDI: in-depth interview
IMAGINE: Interactive Maternal Group for Information and Emotional Support
MB: Mothers and Babies
REDCap: Research Electronic Data Capture
